# Effect of Carperitide on Clinical Outcomes in Patients With Heart Failure: A Systematic Review and Meta-Analysis

**DOI:** 10.7759/cureus.89596

**Published:** 2025-08-08

**Authors:** Zeeshan Ayaz, Loiy Naser Alsarkhi, Anurag Rawat, Yousef Aqel, Maryam Mazhar, Sandipkumar S Chaudhari, Mohammed Qasim Rauf, Neelum Ali

**Affiliations:** 1 Medicine, Rehman Medical Institute, Peshawar, PAK; 2 Internal Medicine, Hamad Medical Corporation, Doha, QAT; 3 Interventional Cardiology, Himalayan Institute of Medical Sciences, Dehradun, IND; 4 Medicine, Hamad Medical Corporation, Doha, QAT; 5 Department of Medicine, Services Institute of Medical Sciences, Lahore, PAK; 6 Cardiothoracic Surgery, University of Alabama at Birmingham, Birmingham, USA; 7 Family Medicine, University of North Dakota School of Medicine and Health Sciences, Fargo, USA; 8 Trauma and Orthopaedics, The Hillingdon Hospitals NHS Foundation Trust, London, GBR; 9 Internal Medicine, University of Health Sciences, Lahore, PAK

**Keywords:** atrial natriuretic peptide, carperitide, heart failure, meta-analysis, mortality

## Abstract

Heart failure remains a leading global health burden affecting over 64 million individuals worldwide, with limited effective acute management strategies. Carperitide, a recombinant form of human atrial natriuretic peptide, has been primarily used in Japan for acute heart failure treatment, but its clinical efficacy remains controversial. This systematic review and meta-analysis aimed to evaluate the clinical outcomes of carperitide in heart failure patients. A comprehensive literature search was conducted across PubMed, Embase, Web of Science, and Cochrane Central Register of Controlled Trials (CENTRAL) from inception to July 2025, using terms related to carperitide and heart failure. Studies involving adult heart failure patients receiving carperitide that reported cardiovascular mortality, all-cause mortality, or heart failure-related hospitalization were included. Six studies met inclusion criteria, comprising three observational studies, two randomized controlled trials, and one secondary analysis, published between 2008 and 2025. Meta-analysis was performed using RevMan 4.5.1 (The Cochrane Collaboration) with random-effects models. The pooled analysis of four studies examining all-cause mortality demonstrated no significant difference between carperitide and control groups (risk ratio (RR): 1.02, 95% confidence interval (CI): 0.63-1.66), with substantial heterogeneity (I² = 66%). Similarly, hospitalization due to heart failure showed no significant difference (RR: 0.98, 95% CI: 0.85-1.14) with no heterogeneity (I² = 0%). Composite outcomes of hospitalization and death also revealed no significant benefit (RR: 0.98, 95% CI: 0.85-1.14). This meta-analysis provides evidence that carperitide does not significantly improve clinical outcomes in heart failure patients, challenging its widespread use and supporting the need for evidence-based therapeutic alternatives in heart failure management.

## Introduction and background

Heart failure (HF) remains a major global health burden, affecting over 64 million individuals worldwide and contributing substantially to morbidity, hospitalizations, and healthcare expenditures [1]. Despite advances in pharmacological and device-based therapies, the prognosis of HF remains poor, particularly in patients with acute decompensated heart failure (ADHF) [2]. While heart failure encompasses both heart failure with reduced ejection fraction (HFrEF) and heart failure with preserved ejection fraction (HFpEF), the majority of carperitide research has focused on acute decompensated presentations across both phenotypes, though treatment response may vary between these populations [3]. Carperitide is primarily utilized in acute hospital settings for short-term hemodynamic stabilization, typically administered as a continuous intravenous infusion at doses ranging from 0.025-0.1 μg/kg/min for 24-72 hours, rather than for chronic outpatient management [2]. The need for effective acute management strategies that improve both symptomatic relief and long-term outcomes remains unmet.

Carperitide, a recombinant form of human atrial natriuretic peptide (ANP), has been developed and is primarily used in Japan for the treatment of acute heart failure [3]. It exerts its therapeutic effects through multiple mechanisms, including vasodilation, natriuresis, diuresis, inhibition of the renin-angiotensin-aldosterone system (RAAS), and suppression of sympathetic nervous system activity [4]. These effects collectively contribute to the reduction of cardiac preload and afterload, improvement in hemodynamics, and enhancement of renal function. Unlike traditional loop diuretics - which may worsen renal function and stimulate neurohormonal activation - carperitide has been proposed as a more physiological alternative that maintains hemodynamic stability without significantly compromising renal perfusion [5]. 

The clinical development of carperitide has been primarily concentrated in Japan, where it received regulatory approval for the treatment of acute heart failure in 1995 [6]. Despite early promising studies demonstrating acute hemodynamic improvements, carperitide's adoption has remained geographically limited due to mixed results from subsequent trials, including some studies reporting increased mortality concerns, and the absence of large-scale randomized controlled trials demonstrating clear long-term clinical benefits that would satisfy regulatory requirements in Western countries. This systematic review addresses this evidence gap by comprehensively evaluating both the acute hemodynamic effects and long-term clinical outcomes of carperitide, including safety parameters such as hypotension and renal adverse effects, building upon prior narrative reviews to provide the first comprehensive meta-analytic synthesis of available evidence

Initial studies demonstrated promising hemodynamic effects, with 82% of patients assessed as clinically improved following carperitide treatment [6]. However, translating these hemodynamic benefits into meaningful clinical outcomes has required further investigation. Carperitide’s mechanism of action involves binding to natriuretic peptide receptors, leading to increased cyclic guanosine monophosphate (cGMP) production and subsequent vasodilation and natriuresis [7].

Clinical trials evaluating carperitide have yielded mixed results. While some studies have demonstrated improvements in hemodynamic parameters and symptom relief, others have raised concerns regarding its impact on hard clinical endpoints such as mortality and rehospitalization [8]. For example, some studies have reported a significant association between carperitide use and increased in-hospital mortality among patients with acute heart failure [9], whereas other research suggests that low-dose carperitide is significantly associated with reduced cardiovascular and all-cause mortality within one year after admission [10].

Given the conflicting evidence and the need for evidence-based treatment recommendations, a comprehensive systematic review and meta-analysis of carperitide’s clinical outcomes in heart failure patients is essential. Such an analysis would provide valuable insights into the drug’s efficacy, safety profile, and potential role in contemporary heart failure management, ultimately informing clinical decision-making and guiding future research directions in this critical area of cardiovascular medicine.

## Review

Methodology 

Literature Search and Search Strategy 

A comprehensive literature search was conducted across multiple electronic databases, including PubMed, Embase, Web of Science, and the Cochrane Central Register of Controlled Trials (CENTRAL), from database inception to July 5, 2025. The search strategy incorporated both Medical Subject Headings (MeSH) and free-text terms related to “Carperitide” and “heart failure.” Keywords included but were not limited to: “Carperitide,” “atrial natriuretic peptide,” “ANP,” “heart failure,” “cardiac failure,” “acute decompensated heart failure,” “cardiovascular mortality,” “hospitalization,” and “all-cause mortality.” Boolean operators (AND, OR) were used to combine terms appropriately. No language restrictions were applied during the initial search. Additionally, the reference lists of all included articles and relevant reviews were manually screened to identify any additional eligible studies. The search strategy was peer-reviewed by a principal investigator independent expert using the PRESS (Peer Review of Electronic Search Strategies) guideline.

Eligibility Criteria and Study Selection 

Studies were included if they met the following criteria: (1) involved adult patients diagnosed with heart failure; (2) evaluated the administration of carperitide, either as monotherapy or in combination with other treatments; (3) reported at least one of the following outcomes-cardiovascular mortality, all-cause mortality, or heart failure-related hospitalization; and (4) were randomized controlled trials (RCTs), cohort studies, or case-control studies. Studies were excluded if they were non-original articles (e.g., reviews, editorials, conference abstracts without full text), animal studies, or lacked sufficient outcome data. 

Two independent reviewers screened all titles and abstracts for relevance. Full texts of potentially eligible studies were retrieved and assessed against the inclusion and exclusion criteria. The final list of included studies was compared, and any disagreements were resolved through discussion or, when necessary, adjudication by a third reviewer.

Data Extraction and Quality Assessment 

Data extraction was independently performed by two reviewers using a standardized data collection form. The following information was extracted from each included study: first author’s name, publication year, country, study design, treatment groups, sample size, duration of follow-up, and reported clinical outcomes. Specifically, data on cardiovascular mortality, all-cause mortality, and hospitalization due to heart failure were extracted. Discrepancies were resolved through discussion or adjudication by a third reviewer.

The risk of bias in randomized controlled trials was assessed using the Cochrane Risk of Bias 2.0 tool [11], while observational studies were evaluated using the Newcastle-Ottawa Scale (NOS) [12]. Discrepancies in scoring were discussed and resolved by consensus. 

Statistical Analysis 

A meta-analysis was performed using RevMan Version 4.5.1 (The Cochrane Collaboration). For dichotomous outcomes, risk ratios (RRs) with corresponding 95% confidence intervals (CIs) were calculated. A random-effects model was used to account for anticipated heterogeneity across studies. Statistical heterogeneity was assessed using the I² statistic, with values >50% indicating substantial heterogeneity. Subgroup analyses and sensitivity analyses were conducted where appropriate. Publication bias was not evaluated as number of included studies was less than 10.

Results 

**Figure 1:**
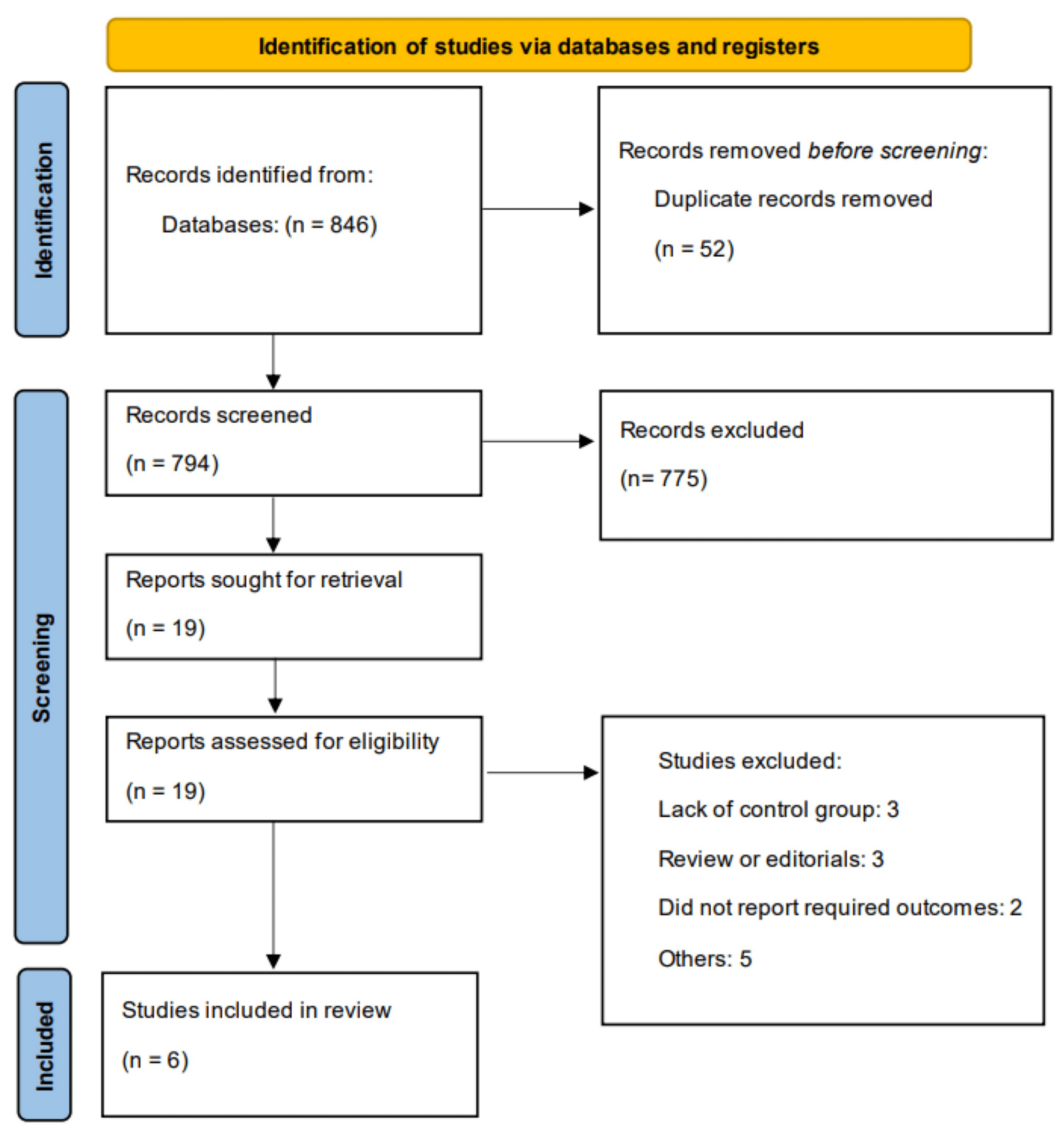
Preferred Reporting Items for Systematic Reviews and Meta-Analyses (PRISMA) flowchart of study selection process

A total of 846 records were identified through systematic searches of online databases. After title and abstract screening, 19 studies were selected for full-text review. Following detailed assessment, six studies met the inclusion criteria and were included in the final meta-analysis. Of these, three were observational studies, two were RCTs, and one was a secondary analysis of an RCT. The Preferred Reporting Items for Systematic Reviews and Meta-Analyses (PRISMA) flow diagram (Figure 1) outlines the detailed study selection process. The characteristics of the included studies are summarized in Table 1. All included studies were published between 2008 and 2025. In terms of follow-up duration, two studies reported in-hospital outcomes, two studies followed participants for up to one year, and two studies reported outcomes beyond one year. Table 2 presents quality assessment of the included RCTs.

**Table 1:** Characteristics of included studies RCT: Randomized-control trial; NR: Not reported

Author	Year	Study Design	Groups	Sample Size	Follow-up	Age	Males	Hypertension	Diabetes
Hata et al. [13]	2008	RCT	Carperitide	26	1.5 Years	67.4	14	NR	7
			Control	23		68.9	18		9
Honda et al. [14]	2024	RCT	Carperitide	125	2 Years	NR	NR	NR	NR
			Control	122					
Matsue et al. [9]	2015	Retrospective	Carperitide	369	In hospital	77.6	188	235	131
			Control	369		77.9	185	245	120
Nogi et al. [10]	2022	Retrospective	Carperitide	1337	1 Year	78	760	936	505
			Control	1098		80	587	719	394
Ogiso et al. [15]	2017	Retrospective	Carperitide	177	In hospital	76	102	124	50
			Control	177		75	106	129	59
Suzuki et al. [16]	2025	RCT	Carperitide	168	1 Years	NR	NR	NR	NR
			Control	168					

**Table 2:** Quality assessment of included observational studies

Author	Comparison	Selection	Assessment	Overall Grade
Matsue et al. [9]	3	1	3	Good
Nogi et al. [10]	4	2	3	Good
Ogiso et al. [13]	3	2	2	Good

*Meta-Analysis of Outcomes* 

**Figure 2:**
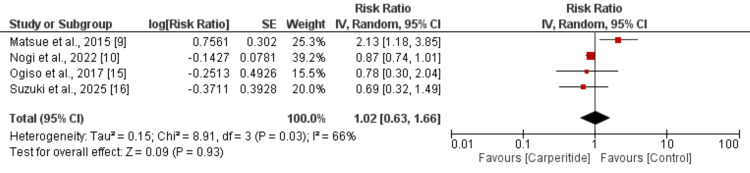
Comparison of mortality between two groups References [9,10,15,16]

All-cause mortality: Four studies reported data on all-cause mortality comparing patients who received carperitide with those who did not. The results are presented in Figure 2. The pooled analysis demonstrated no significant difference in all-cause mortality between the two groups (risk ratio [RR]: 1.02, 95% confidence interval [CI]: 0.63 to 1.66). However, there was substantial heterogeneity among the included studies (I² = 66%), indicating variability in the effect estimates across studies. 

**Figure 3:**
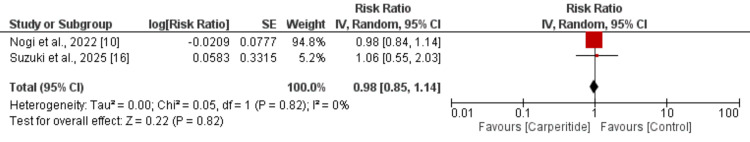
Comparison of hospitalization due to heart failure between two groups References [10,16]

Hospitalization due to heart failure: Hospitalization due to heart failure was assessed in two studies comparing patients treated with carperitide to those who did not receive it. As illustrated in Figure 3, the combined analysis revealed no statistically significant difference in hospitalization rates between the two groups (RR: 0.98, 95% CI: 0.85-1.14). Notably, there was no evidence of heterogeneity across these studies, with an I² value of 0%, indicating consistent findings.

**Figure 4:**
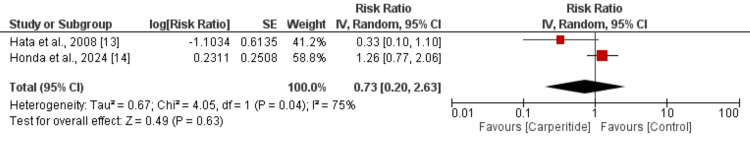
Comparison of composite outcomes between two groups References [13,14]

Combination of hospitalization and death: Two studies reported a composite outcome of hospitalization and death, providing combined effect estimates for patients who received carperitide versus those who did not. The results of the pooled analysis are presented in Figure 4. The analysis showed no significant difference between the carperitide and non-carperitide groups (RR: 0.98, 95% CI: 0.85 to 1.14). There was high heterogeneity observed between the studies (I² = 75%).

Discussion 

This meta-analysis represents the most comprehensive systematic review to date evaluating the clinical outcomes of carperitide in patients with heart failure. The findings demonstrate that carperitide does not significantly improve all-cause mortality, heart failure hospitalization rates, or composite outcomes when compared with standard care or alternative vasodilator therapies. These results have important implications for clinical practice and challenge the widespread use of carperitide in certain healthcare systems.

The pooled analysis of four studies assessing all-cause mortality revealed no significant difference between carperitide and control groups (RR: 1.02, 95% CI: 0.63-1.66). This finding is particularly noteworthy given the theoretical benefits of ANP therapy in heart failure management. ANP exhibits multiple cardioprotective properties, including natriuresis, vasodilation, and inhibition of the renin-angiotensin-aldosterone system, which would be expected to translate into improved clinical outcomes [17,18]. However, our results align with recent concerns raised by several observational studies questioning carperitide’s clinical effectiveness and safety profile [8,15].

The substantial heterogeneity observed in mortality outcomes (I² = 66%) indicates significant variability in treatment effects across different patient populations and clinical settings. This heterogeneity may be due to differences in patient selection criteria, dosing regimens, concomitant therapies, or baseline disease severity among the included studies. Previous research has suggested that carperitide’s efficacy may be dose-dependent, with low-dose therapy potentially offering benefits not seen with very low or higher doses [10]. These findings underscore the complexity of carperitide’s clinical effects and highlight the need for more standardized approaches to patient selection and dosing.

Our findings are consistent with broader concerns regarding vasodilator therapy in acute heart failure. A recent comprehensive meta-analysis of vasodilators in acute heart failure, which included 46 randomized controlled trials with 28,374 patients, found no reduction in all-cause mortality (RR: 0.95, 95% CI: 0.87-1.04) [19]. The 2021 European Society of Cardiology guidelines have downgraded the recommendation for vasodilators due to recent studies failing to demonstrate benefit from this drug class [20]. This broader context suggests that our findings regarding carperitide may reflect fundamental limitations in the vasodilator approach to acute heart failure rather than drug-specific shortcomings.

The lack of observed benefit with carperitide stands in contrast to the established efficacy of other heart failure therapies. Contemporary network meta-analyses have demonstrated significant mortality benefits from combinations of angiotensin receptor-neprilysin inhibitors, beta-blockers, mineralocorticoid receptor antagonists, and sodium-glucose cotransporter 2 (SGLT2) inhibitors [21]. This disparity underscores the importance of evidence-based therapeutic choices and raises questions about the continued use of carperitide as a first-line therapy.

The potential for carperitide-induced hypotension is particularly concerning in the acute heart failure setting, where hemodynamic instability may lead to organ hypoperfusion and worse outcomes. Research has identified hypotension within 12 hours and baseline renal dysfunction as independent predictors of worsening renal function in patients treated with low-dose carperitide [22]. These findings emphasize the importance of careful patient selection and close monitoring when considering carperitide therapy.

Several limitations should be acknowledged when interpreting the present study findings. First, the substantial heterogeneity in mortality outcomes limits the generalizability of the results across all patient populations. Second, most included studies were observational or had relatively small sample sizes, potentially limiting the power to detect meaningful differences in clinical outcomes. Third, variations in carperitide dosing, therapy duration, and concomitant treatments across studies may have influenced the observed effects. Additionally, we did not perform publication bias as number of included studies was less than 10. Lastly, we were unable to perform subgroup analyses or meta-regression due to the limited number of studies available for each outcome.

Future research should focus on well-designed randomized controlled trials to determine optimal dosing strategies and identify patient subgroups that may benefit from carperitide therapy. Given the dose-dependent effects suggested by some studies, systematic evaluation of different dosing regimens is essential to understanding carperitide’s therapeutic potential. Additionally, head-to-head trials comparing carperitide with established heart failure therapies would offer valuable insights into its relative effectiveness.

Clinical Practice Implications

Based on our findings, carperitide should not be considered as first-line therapy for acute heart failure given the lack of mortality or hospitalization benefits and potential safety concerns. However, clinicians may consider its use in select scenarios where conventional diuretics are contraindicated or have failed, particularly in patients with severe renal dysfunction where preserving renal perfusion is critical, though close hemodynamic monitoring is essential.

Healthcare institutions should develop specific protocols for identifying patients currently receiving carperitide who may benefit from alternative approaches, while establishing clear criteria for the limited circumstances where carperitide use might still be justified pending further definitive research

## Conclusions

This comprehensive systematic review and meta-analysis provides the most robust evidence to date regarding carperitide's clinical effectiveness in heart failure management. Our findings conclusively demonstrate that carperitide does not confer significant benefits for all-cause mortality, heart failure hospitalizations, or composite clinical outcomes compared to standard care. Despite theoretical advantages of atrial natriuretic peptide therapy, including vasodilation, natriuresis, and neurohormonal modulation, these physiological effects do not translate into meaningful clinical improvements. The substantial heterogeneity observed in mortality outcomes highlights the complexity of carperitide's effects across different patient populations and clinical settings. These results challenge current practice patterns in healthcare systems where carperitide is extensively utilized and underscore the critical importance of evidence-based therapeutic decision-making in heart failure management. Future research should focus on identifying specific patient subgroups who might benefit from carperitide therapy and establishing optimal dosing strategies through well-designed randomized controlled trials to definitively determine its clinical utility.

## References

[1] Savarese G, Becher PM, Lund LH, Seferovic P, Rosano GM, Coats AJ (2023). Global burden of heart failure: a comprehensive and updated review of epidemiology. Cardiovasc Res.

[2] Aronson D, Krum H (2012). Novel therapies in acute and chronic heart failure. Pharmacol Ther.

[3] Matsumoto S, Nakazawa G, Ohno Y (2020). Efficacy of exogenous atrial natriuretic peptide in patients with heart failure with preserved ejection fraction: deficiency of atrial natriuretic peptide and replacement therapy. ESC Heart Fail.

[4] Mitaka C, Kudo T, Haraguchi G, Tomita M (2011). Cardiovascular and renal effects of carperitide and nesiritide in cardiovascular surgery patients: a systematic review and meta-analysis. Crit Care.

[5] Suwa M, Seino Y, Nomachi Y, Matsuki S, Funahashi K (2005). Multicenter prospective investigation on efficacy and safety of carperitide for acute heart failure in the 'real world' of therapy. Circ J.

[6] Sandefur CC, Jialal I (2025). Atrial natriuretic peptide. StatPearls [Internet].

[7] Asanuma H, Sanada S, Asakura M (2014). Carperitide induces coronary vasodilation and limits infarct size in canine ischemic hearts: role of NO. Hypertens Res.

[8] Nagai T, Iwakami N, Nakai M (2019). Effect of intravenous carperitide versus nitrates as first-line vasodilators on in-hospital outcomes in hospitalized patients with acute heart failure: insight from a nationwide claim-based database. Int J Cardiol.

[9] Matsue Y, Kagiyama N, Yoshida K (2015). Carperitide is associated with increased in-hospital mortality in acute heart failure: a propensity score-matched analysis. J Card Fail.

[10] Nogi K, Ueda T, Matsue Y (2022). Effect of carperitide on the 1 year prognosis of patients with acute decompensated heart failure. ESC Heart Fail.

[11] Minozzi S, Cinquini M, Gianola S, Gonzalez-Lorenzo M, Banzi R (2020). The revised Cochrane risk of bias tool for randomized trials (RoB 2) showed low interrater reliability and challenges in its application. J Clin Epidemiol.

[12] Wells G, Shea B, O’connell D, Peterson J, Welch V, Losos M, Tugwell P (2014). Newcastle-Ottawa quality assessment scale cohort studies. University of Ottawa.

[13] Hata N, Seino Y, Tsutamoto T (2008). Effects of carperitide on the long-term prognosis of patients with acute decompensated chronic heart failure: the PROTECT multicenter randomized controlled study. Circ J.

[14] Honda S, Nagai T, Honda Y (2025). Effect of low-dose administration of carperitide for acute heart failure: the LASCAR-AHF trial. Eur Heart J Acute Cardiovasc Care.

[15] Ogiso M, Isogai T, Okabe Y, Ito K, Tsuji M, Tanaka H (2017). Effect of carperitide on in-hospital mortality of patients admitted for heart failure: propensity score analyses. Heart Vessels.

[16] Suzuki A, Shiga T (2025). Effect of Carperitide on clinical outcomes in patients with acute decompensated heart failure: a subanalysis of the HIJ-HF II study. Rinsho Yakuri.

[17] Yancy CW, Jessup M, Bozkurt B (2017). 2017 ACC/AHA/HFSA focused update of the 2013 ACCF/AHA guideline for the management of heart failure: a report of the American College of Cardiology/American Heart Association Task Force on Clinical Practice Guidelines and the Heart Failure Society of America. J Am Coll Cardiol.

[18] Potter LR, Abbey-Hosch S, Dickey DM (2006). Natriuretic peptides, their receptors, and cyclic guanosine monophosphate-dependent signaling functions. Endocr Rev.

[19] Lukoschewitz JD, Miger KC, Olesen AS (2024). Vasodilators for acute heart failure—a systematic review with meta-analysis. NEJM Evid.

[20] McDonagh TA, Metra M, Adamo M (2021). 2021 ESC Guidelines for the diagnosis and treatment of acute and chronic heart failure. Eur Heart J.

[21] Tromp J, Ouwerkerk W, van Veldhuisen DJ (2022). A systematic review and network meta-analysis of pharmacological treatment of heart failure with reduced ejection fraction. JACC Heart Fail.

[22] Nomura F, Kurobe N, Mori Y, Hikita A, Kawai M, Suwa M, Okutani Y (2008). Multicenter prospective investigation on efficacy and safety of carperitide as a first-line drug for acute heart failure syndrome with preserved blood pressure: COMPASS: Carperitide Effects Observed Through Monitoring Dyspnea in Acute Decompensated Heart Failure Study. Circ J.

